# Knowledge, Attitudes and Practices Related to Antimicrobial Use and Resistance Among Livestock Sector Stakeholders in Seven Former Soviet Countries: A Multi-Country Regional Analysis

**DOI:** 10.3390/antibiotics15040384

**Published:** 2026-04-09

**Authors:** Dora Kovacs, Eran Raizman, Anne Deckert, Chichak Aliyeva, Dragan Angelovski, Zaruhi Beglaryan, Duriya Charypkhan, Natalia Ciria, Tolibjon Khakimov, Maripa Kichinebatyrova, Elvira Maratova, Tamas Nagy, Anna Sargsyan, Oksana Yurchenko, Daniel Beltran-Alcrudo

**Affiliations:** 1 Food and Agriculture Organization of the United Nations, Regional Office for Europe and Central Asia, H-1054 Budapest, Hungary; eran.raizman@fao.org (E.R.); daniel.beltranalcrudo@fao.org (D.B.-A.); 2 Department of Population Medicine, Ontario Veterinary College, University of Guelph, Guelph, ON N1G 2W1, Canada; adeckert@uoguelph.ca; 3 Hasvet School of Veterinary Medicine, Khazar University, Baku AZ1096, Azerbaijan; chichak.aliyeva@khazar.org; 4 Food and Agriculture Organization of the United Nations, Representation in Georgia, Tbilisi 0159, Georgia; 5 Food and Agriculture Organization of the United Nations, Representation in Armenia, Yerevan 0010, Armenia; zaruhi.beglaryan@fao.org; 6 Food and Agriculture Organization of the United Nations, Representation in Kazakhstan, Astana Z05M0M3, Kazakhstan; duriya.charypkhan@fao.org; 7 Department of Animal Health and Anatomy, Autonomous University of Barcelona, 08193 Barcelona, Spain; natalia.ciria@uab.cat; 8 Food and Agriculture Organization of the United Nations, Representation in Tajikistan, Dushanbe 734025, Tajikistan; tolibjon.khakimov@fao.org; 9 Food and Agriculture Organization of the United Nations, Representation in Kyrgyzstan, Bishkek 720017, Kyrgyzstan; marina.kichinebatyrova@fao.org (M.K.); elviramaratovaa@gmail.com (E.M.); 10 Gyermelyi Holding Zrt., H-2821 Gyermely, Hungary; drnagyvet@gmail.com; 11 Strategic Development Agency, Yerevan 0070, Armenia; a.sargsyan@sdaoffice.com; 12 Food and Agriculture Organization of the United Nations, Representation in Ukraine, Kyiv 01021, Ukraine; oksana.yurchenko@fao.org

**Keywords:** antimicrobial resistance, antimicrobial use, former Soviet countries, low- and middle-income countries, knowledge, attitudes and practices, livestock

## Abstract

**Background/Objectives:** Antimicrobial resistance (AMR) is one of the greatest health threats affecting humans, animals and the environment. Antimicrobial use (AMU) in the livestock sector contributes to the development and spread of AMR, highlighting the need to understand the current situation, to target knowledge gaps and non-prudent practices with tailored interventions, and improve antimicrobial stewardship. This is especially important in low- and middle-income countries (LMICs), where data on AMU and AMR are currently limited. This study assessed knowledge, attitudes and practices (KAP) among farmers, veterinarians, veterinary pharmacy personnel and feed mill personnel related to AMU (particularly considering the use of antibiotics) and AMR in seven former Soviet countries, Armenia, Azerbaijan, Georgia, Kazakhstan, Kyrgyzstan, Tajikistan and Ukraine. **Methods:** Face-to-face interviews were conducted between 2020 and 2025 with 3012 participants, with results analyzed using an aggregated regional approach. **Results:** The interviews revealed common regional knowledge gaps and practices among livestock sector stakeholders related to antimicrobials, AMR, antimicrobial residues, and prudent AMU. Non-prudent practices, such as the purchase of antimicrobials without a prescription, the use of antimicrobials as growth promoters, the inappropriate disposal of antimicrobials, and the frequent use of highest priority critically important antimicrobials (HPCIAs) were reported. Another factor that may hinder prudent AMU was the limited access of veterinarians to diagnostic laboratories. **Conclusions:** Despite significant global efforts to tackle AMR, there is an ongoing need to address knowledge gaps and non-prudent practices of livestock sector stakeholders in former Soviet countries. The findings highlight the importance of antimicrobial stewardship interventions that address system-level drivers of improper AMU beyond stakeholder trainings.

## 1. Introduction

The use of antimicrobials in veterinary medicine has various benefits for animal health and welfare, sustainable food production, agricultural livelihoods, and, by reducing the risk of epidemics and foodborne diseases, for public health. On the other hand, the inappropriate use of antimicrobials, particularly for non-therapeutic purposes in food-producing animals, contributes significantly to the global development and spread of antimicrobial resistance (AMR), negatively affecting the availability of effective treatment options for both humans and animals [1]. Low- and middle-income countries (LMICs) may have a greater burden of AMR compared to high-income countries (HICs). On one hand, this may be due to higher infection rates in LMICs [2,3], but other factors, such as poor hygiene and sanitation, inadequate waste management, limited access to quality healthcare, lax regulations on antimicrobial use (AMU) or lack of enforcement, the presence and use of counterfeit antimicrobials, and poor medical, veterinary, livestock, and public sector knowledge on optimal AMU, leading to cases of misuse, can accelerate the spread of AMR [3,4]. The lack of laboratory diagnostic capacity for microbiological tests and the disproportionate availability of different antimicrobial classes can pose additional challenges for antimicrobial stewardship [4]. These factors may affect both human and animal healthcare. In addition, living in close contact with animals, which is more common in LMICs [5], may also favour the spread of resistant microorganisms between humans and animals.

After the collapse of the Soviet Union, the newly independent countries experienced political, social, and economic crises [6,7], followed by strong economic rebounds in only some of them [8]. Currently, former Soviet countries are mainly ranked by the World Bank as either upper middle-income countries (Armenia, Azerbaijan, Belarus, Georgia, Kazakhstan, Republic of Moldova, Turkmenistan and Ukraine) or LMICs (Kyrgyzstan, Tajikistan and Uzbekistan). Only the Baltic states (Estonia, Latvia and Lithuania) and the Russian Federation are categorized as HICs [9]. As LMICs, many of the former Soviet countries may face a high burden of AMR, although it is difficult to precisely estimate the magnitude of AMR in LMICs due to the lack of well-structured and comprehensive surveillance systems. This knowledge gap is aimed to be filled by the Global Antimicrobial Resistance and Use Surveillance System (GLASS) project of the World Health Organization (WHO) for human health [10], and by the International Food and Agriculture Organization of the United Nations (FAO) Antimicrobial Resistance Monitoring (InFARM) system for the animal health side [11]. LMICs also commonly lack AMU surveillance systems for both humans and animals [3]. Despite the lack of comprehensive surveillance systems, studies from former Soviet countries indicate alarming rates of resistant and multidrug-resistant (MDR) infections in human healthcare, including resistance to highest priority critically important antimicrobials (HPCIA) according to the WHO categorization, posing a significant challenge to disease treatment [12,13,14,15,16,17,18,19,20,21].

When it comes to AMR data from the animal health sector, a high prevalence of resistance has also been reported from former Soviet countries. For example, high rates of resistance (above 70%) to tetracycline and ciprofloxacin were detected in a Georgian study with *Campylobacter coli* and *C. jejuni* isolates from poultry [17]. In studies from Kazakhstan, *Staphylococcus aureus* isolates obtained from milk samples of cows with clinical and subclinical mastitis showed the highest levels of resistance to tetracyclines and beta-lactam antibiotics (up to 100%) [22,23], and resistance was also common against fluoroquinolones (above 90%) in isolates from clinical mastitis cases [23]. Other studies from Kazakhstan found high levels of resistance to tetracyclines [24,25] and aminoglycosides [25] in *Salmonella* spp. isolates (up to more than 50%), and to beta-lactams in *S. aureus* (95%) [24], originating from samples of animals and foods of animal origin [24,25]. MDR *Escherichia coli* isolates were also found in contaminated cheese samples from Kazakhstan [26]. In studies from dairy farms in Ukraine, resistance to beta-lactam antibiotics was commonly observed in *E. coli* and *S. aureus* isolates, while tetracycline resistance was frequently detected in *Streptococcus agalactiae* (up to 80%) [27,28]. Another study from Ukraine showed resistance in *E. coli* isolates from wild waterfowl that had never been treated with antimicrobials [29].

Confirming the growing commitment to tackling the threat of resistance, most former Soviet countries have developed National Action Plans (NAPs) on AMR [30], although their implementation remains challenging in many settings, especially in LMICs [12]. FAO, together with WHO, the World Organization for Animal Health (WOAH), and the United Nations Environment Programme (UNEP), collectively referred to as the Quadripartite, supports countries in implementing their NAPs on AMR. FAO developed the Progressive Management Pathway for Antimicrobial Resistance (FAO-PMP-AMR) methodology, which aids countries in assessing their AMR NAP implementation progress and in prioritizing actions to be taken to tackle AMR (https://www.fao.org/antimicrobial-resistance/resources/tools/fao-pmp-amr/en/ (accessed on 3 February 2026)). The tool has already been deployed in more than 40 countries across the globe, including former Soviet countries. The outcomes of the FAO-PMP-AMR assessments have shown, among other areas, the need to understand AMU practices in the livestock sector.

This study was conducted as part of FAO’s regional work on AMR, in line with FAO-PMP-AMR outcomes, to assess the knowledge, attitudes and practices (KAP) of stakeholders in the livestock sector (farmers, field veterinarians, veterinary pharmacy personnel and feed mill personnel) related to AMU and AMR in seven former Soviet countries: Armenia, Azerbaijan, Georgia, Kazakhstan, Kyrgyzstan, Tajikistan and Ukraine. While data have been published on human AMU in former Soviet countries [31], there remains a lack of information on AMU patterns in the livestock sector. This study addresses this knowledge gap and provides findings that can inform the design of targeted trainings, awareness-raising, and policy interventions to support prudent AMU across the Europe and Central Asia region. Antimicrobials covered by the surveys and discussed in the manuscript refer only to antibacterials.

## 2. Results

This manuscript presents the summary results (aggregated data) for Armenia, Azerbaijan, Georgia, Kazakhstan, Kyrgyzstan, Tajikistan and Ukraine. This aggregated approach was used to identify systemic patterns in AMU practices, knowledge gaps, access to veterinary services and diagnostics, prescription use, and stewardship challenges that are shared across countries with a common post-Soviet institutional legacy and comparable livestock production and veterinary service structures. While acknowledging national differences, the regional synthesis enables a systems-level interpretation of AMU drivers that would not be apparent from country-specific analyses alone. Country-specific results are available in the detailed national reports on FAO’s website (https://www.fao.org/antimicrobial-resistance/resources/publications-archive/en/ (accessed on 3 February 2026)) [32,33,34,35,36].

### 2.1. Demographics

A total of 1945 farmers, 506 veterinarians, 486 veterinary pharmacy personnel and 75 feed mill personnel were surveyed in this study. The number of interviews conducted is shown in Table 1. Farmers were requested to complete species-specific surveys for all animal species that they had on their farms for income. For example, a farmer who raised both chickens and cattle for sale completed both the chicken and cattle questionnaires. Therefore, the total number of species-specific interviews is greater than the total number of farmers surveyed in the study. Farmers who primarily raised livestock for their own consumption completed the backyard questionnaire. Farmers, veterinarians and veterinary pharmacy personnel were evenly distributed among age groups of 25–40 years, 41–55 years, and over 55 years, while only 2% of farmers, 4% of veterinarians, and 3% of veterinary pharmacy personnel were younger than 25 years. Feed mill personnel were mainly between 25 and 40 years (33%) or 41–55 years (56%) of age. Most respondents from all stakeholder groups were male (77% of farmers, 88% of veterinarians, 80% of veterinary pharmacy personnel, and 80% of feed mill personnel). The farmers, veterinary pharmacy personnel, and feed mill personnel interviewed were mainly the manager or owner of the farm (87%), the pharmacy (88%), or the feed mill (59%), respectively. The median years of experience in their profession were 18 years among farmers, 20 years among veterinarians, and 13 years among veterinary pharmacy personnel. More than half (56%) of the surveyed farmers did not have any previous education on animal health, animal husbandry, pharmacology, or other agriculture-related areas. A considerable number of veterinarians (29%) reported having income from sources other than their veterinary practice, usually from livestock farming.

### 2.2. Interviews with Farmers

#### 2.2.1. Practices

It was not common among the surveyed farmers to keep records related to animal health. Approximately one-third had records about veterinary visits (36%), vaccinations (36%), animal medicines purchased (35%), treatments (33%) and prescriptions (32%). Around half of the participants (54%) did not keep records for any of these activities. Slightly more farmers kept records on animal births (46%) and mortality (39%) on their farm.

In terms of on-farm hygiene and biosecurity measures, the most commonly reported good biosecurity practices included keeping wild animals away from the farm, having rodent and pest control measures, and isolating sick and new animals. In addition, farmers rarely shared equipment and breeding stock with other farmers. Chicken farmers also ensured good ventilation in the barns, and dairy farmers used pre- and post-milking teat dips, gave colostrum to calves, and milked sick cows last. Among beekeepers, cleaning the hives before introducing a new colony was the most frequently practised good biosecurity measure. On the other hand, regardless of the species housed, it was rare for farmers to disinfect vehicles at the farm entrance, to register visitors, and to provide them with protective clothes and boots.

Around half of the surveyed farmers (52%, 1007 participants) reported using antimicrobials in their animals. The following section details the responses of these 1007 antimicrobial users. Farmers predominantly purchased antimicrobials from veterinary pharmacies (83% always or often), and less commonly from wholesalers (28%), private veterinarians (22%) or government veterinarians (18%). The majority never bought antimicrobials from outside of the country (95%), other farmers (94%), local market (82%), or human pharmacies (68%). When buying antimicrobials, 60% of farmers reported always having a prescription, while 19% often and 6% occasionally had one. The rest of the farmers either rarely (5%) or never (10%) had a prescription for the purchase.

The decision to use antimicrobials was primarily made by farm owners (always or often on 48% of farms), followed by government veterinarians (40%), company veterinarians (38%) and private veterinarians (35%). In terms of advice on antimicrobial drug choice, treatment duration and dose, farmers most commonly turned to veterinary pharmacists (59% always or often), followed by government veterinarians (44%), private veterinarians (38%) and company veterinarians (35%). They also commonly relied on information found on the product label (39%) and on their previous experience (38%). Information from the internet or advice from other farmers, friends or family was less frequently considered. Veterinary laboratory results were never used for this purpose by 62% of farmers. Of note, farmers could choose more than one option for these questions, i.e., indicating that they usually received decisions and advice from multiple sources. Almost all farmers (91%) reported following the advice received about antimicrobial treatment dose and duration, while only 7% reported treatments with either a lower dose or for a shorter duration than advised. This finding was surprising, given that 47% of farmers believed that antimicrobial treatment can be stopped when the animal’s clinical signs improve (Table 2).

In terms of handling expired antimicrobials, farmers most commonly threw them in the garbage (53%). This practice was similarly common, regardless of whether farmers were aware that freely discarded antimicrobials may have a negative effect on the environment (Table 2). Only about one quarter (24%) of participants reported either returning expired drugs to the place of purchase or consulting a veterinarian.

#### 2.2.2. Knowledge and Attitudes

Most farmers interviewed (76%) reported knowing what antibiotics are. Among those who reported not knowing what antibiotics were, 14% (63 of 463 farmers) used these drugs in their animals. When asked to choose the definition of these drugs from a list provided, of those who reported knowing what antibiotics were, 44% chose the correct answer (“Medicine that kills bacteria”); however, the majority of them believed that other definitions were also correct. Only 4% of farmers chose the correct definition exclusively. The most frequently selected incorrect definitions were “Medicine that prevents disease” (56%), “Medicine that kills disease” (52%), and “Medicine that kills germs” (50%). Around one-third (27%) selected the definition “Medicine that makes animals grow faster/bigger”. Some farmers also thought that antibiotics are effective against viruses (13%) and parasites (8%).

Farmers who reported using antimicrobials in their animals (1007 respondents) were asked a set of questions about antimicrobials and AMU. Table 2 shows the responses collected in this section of the survey. Farmers in general had good knowledge about the withdrawal period and understood that prevention, including vaccination, and early detection of diseases, are useful in reducing the need for AMU. Many farmers were also aware that if antimicrobials are used too often, they might stop working. These included mainly, but not only, farmers who had heard of AMR prior to the study (62% of participants). On the other hand, many farmers confused the concept of AMR with antimicrobial residues, and a considerable proportion believed that antimicrobial treatment can be stopped when the animal’s symptoms improve. Despite their misconceptions, one quarter (25%) of antimicrobial user farmers were not interested in learning more about these drugs.

**Table 2:** Farmers’ beliefs about antimicrobials and antimicrobial use.

Statements on Antimicrobials and Antimicrobial Use	Farmers’ Replies			# Replies
	Agree	Disagree	Do Not Know	
You can stop giving antibiotics to an animal if their symptoms are improving. (Correct: disagree)	**47%**	*39%*	14%	1002
If antibiotics are given too often, they might stop working. (Correct: agree)	*71%*	**6%**	23%	1000
Giving antibiotics to healthy animals will prevent them from becoming sick in the future. (Correct: disagree)	**28%**	*56%*	16%	999
Using vaccines can prevent the use of antibiotics. (Correct: agree)	*65%*	**12%**	23%	1000
Animals can transmit disease to humans. (Correct: agree)	*90%*	**2%**	8%	1002
Antibiotic use in animals does not affect human health. (Correct: disagree)	**28%**	*51%*	20%	1003
Antibiotics may be freely discarded without having any action/effect on the environment. (Correct: disagree)	**17%**	*57%*	26%	1002
Antibiotic resistance occurs when antibiotics are found in the meat or milk of an animal. (Correct: disagree)	**69%**	*4%*	26%	998
When you use antibiotics, there is a certain number of days you should wait before selling the animals for slaughter, selling eggs, milk, or honey. (Correct: agree)	*85%*	**2%**	13%	1001
With prevention and early detection (of diseases), you can reduce the use of antibiotics. (Correct: agree)	*72%*	**9%**	19%	1000

Correct answers are shown in italics, and misconceptions are shown in bold.

### 2.3. Interviews with Veterinarians

As with farmers, record-keeping was not a common practice among the surveyed veterinarians: 52% did not have any records about the antimicrobials that they sold or prescribed. The names or volume of antimicrobials sold or prescribed per year were recorded by 37% and 31% of respondents, respectively, while even fewer veterinarians kept records about antimicrobial sales or prescriptions per farm or per farm visit. This may have been linked to the fact that many veterinarians did not prescribe or sell antimicrobials to farmers, but were treating the animals directly instead. Only 35% of veterinarians reported always or usually writing a prescription, and 25% always or usually sold antimicrobials to farmers. Sending prescriptions to feed mills for preparing feed containing antimicrobials was even less common, with 80% of veterinarians never doing so. When advising farmers on AMU, 82% of veterinarians always informed the farmer about the withdrawal period, while 10% never did so. This was in agreement with the fact that most farmers were aware of the withdrawal period (Table 2).

Veterinarians predominantly bought antimicrobials from veterinary pharmacies (90% always or usually), and less commonly from human pharmacies (25%) and wholesalers (18%). Of note, veterinarians could choose more than one option for this question, i.e., indicating multiple sources from which they purchased antimicrobials. The majority never bought these drugs from other veterinarians (74%), from the market (83%) or from outside of the country (96%). When farmers had sick animals, the first measure recommended by most veterinarians (88%) was to isolate the sick individuals, while sending samples to a diagnostic laboratory was usually a second-choice measure, and treatment with antimicrobials was mainly the third. Veterinarians’ decision on sending samples for laboratory diagnostic testing depended on whether it was a single case of infection or an outbreak, and whether it was in the herd of a regular client or on a newly visited farm. Most of them would send samples in the case of a disease outbreak in a known (86%) or a new herd (71%), while a lower proportion would do so for single cases (58% for known herds and 44% for new ones). However, only 62% of veterinarians reported having good access to a veterinary diagnostic laboratory, and 16% reported that their clients were not willing to pay for laboratory testing. Interestingly, some veterinarians (8%) believed that laboratory testing was not necessary. When it comes to antimicrobial susceptibility testing (AST), 62% of veterinarians reported that it was available in the laboratory with which they worked. Most, but not all (78%) veterinarians had information on AMR included in their education.

### 2.4. Interviews with Veterinary Pharmacy Personnel

Record-keeping was more common among veterinary pharmacy personnel than among farmers and veterinarians, with 76% having some records about the names, volume or value of antimicrobials sold in their pharmacies. Most frequently, they recorded the names of antimicrobials sold per purchase (57%) or per year (56%), followed by the value of antimicrobials sold per client (53%) or per year (51%), and the volume of antimicrobials sold per purchase (51%) or per year (49%). Records were also kept about the species and production stage (52%) and the number (45%) of animals for which antimicrobials were sold. Veterinary pharmacies primarily obtained antimicrobials from veterinary wholesalers (91% always or usually), and less commonly from human pharmacies (19%). Buying drugs from outside the country and from the market were rare (8% and 6%, respectively). Of note, veterinary pharmacy personnel could choose more than one option for this question, i.e., indicating multiple sources from which they obtained antimicrobials. The main customers of pharmacies were the general public, including farmers (54% of sales on average), followed by private veterinarians (29%) and official veterinarians (20%). In contrast to the information provided by farmers, the majority of veterinary pharmacy personnel reported not requiring a prescription for selling antimicrobials: 44% never required one, and 20% rarely did so. When dispensing antimicrobials, only 69% of veterinary pharmacy personnel provided information about the withdrawal period, mainly obtained from the manufacturer’s instructions of the drugs. Among those veterinary pharmacy personnel who completed veterinary pharmacist training or education (438 of 486 respondents), 75% learned about AMR during their studies.

### 2.5. Interviews with Feed Mill Personnel

From the total of 75 feed mill personnel included in the study, the practice of mixing antimicrobials into the feed, either automatically or upon request, was reported by 31 participants (41%). None of the respondents from Ukraine reported preparing feed containing antimicrobials. The following section describes the findings from those 31 feed mills that prepared feed containing antimicrobials. Good record-keeping practices were observed in the feed mills: 87% of participants had records about either the drug name or the volume of feed sold that contained antimicrobials. They kept these records broken down either by year, by client, by purchase, or by animal species. Most feed mills also had records about the species (81%) and number (68%) of animals for which the medicated feed was used. Feed mills obtained antimicrobials predominantly from veterinary wholesalers (90% always or usually), as well as from outside of the country (45% always or usually). Of note, feed mill personnel could choose more than one option for this question, i.e., indicating multiple sources from which they obtained antimicrobials. The practice of requiring a prescription for selling medicated feed varied among feed mills: 42% always required one, while 35% never did so. When selling medicated feed, the provision of information about the withdrawal period was more consistent, with 71% of participants reporting this practice. They obtained information about the withdrawal period from the user instructions of the drugs, or from the manufacturers and distributors. Most feed mill personnel (71%) reported having learned about AMR during their studies.

### 2.6. Most Used Antimicrobials and Antimicrobial Use Practices

Only a few participants from Georgia provided information about their AMU practices, and none of them specified the actual drugs used. Therefore, this section describes common AMU patterns in Armenia, Azerbaijan, Kazakhstan, Kyrgyzstan, Tajikistan and Ukraine. In the surveys, stakeholders were asked to list the first, second, third, etc., most used, prescribed, or sold antimicrobials in their practice (for farmers, veterinarians, and veterinary pharmacy personnel, respectively). Feed mill personnel reported the antimicrobials that they most commonly mixed into feed. The following section summarizes the drugs listed as the first most used, prescribed, or sold.

The antimicrobials listed by farmers as the most used are shown in Table 3. Overall, oxytetracycline was the most used drug, and it ranked highest on all farm types except for chicken and pig farms. Chicken farmers reported enrofloxacin, while pig farmers reported amoxicillin as the most used substance. Other frequently used drugs included benzylpenicillin alone and in combination with streptomycin or dihydrostreptomycin.

In accordance with the responses of farmers, veterinarians and veterinary pharmacy personnel listed oxytetracycline as the most commonly prescribed or sold antimicrobial drug in their practice (Table 4). The use of amoxicillin and the combination of benzylpenicillin with streptomycin or dihydrostreptomycin were also common. Among HPCIA drugs, both ceftiofur and fluoroquinolones were reported, similar to the responses of farmers. On the other hand, colistin was not among the first 15 most prescribed or sold drugs by veterinarians and veterinary pharmacy personnel, while its use was reported among the top 15 drugs on some farm types.

Farmers used antimicrobials for the treatment and prevention of respiratory and gastrointestinal infections in various animal species, and for mastitis in dairy cattle. Therapeutic use was predominant in ruminants and pigs, and these species were mainly treated with injectable drugs, while both prophylactic and therapeutic use were reported equally by chicken farmers, with drugs commonly administered mixed into the animals’ feed or drinking water. When treatment with antimicrobials did not yield the expected result, farmers usually consulted with a veterinarian. In other cases, farmers repeated the treatment with the same drug or used a different antimicrobial. Only a small proportion (4%, 75 participants) of farmers reported using antimicrobials for growth promotion, and the majority of these were backyard (39%) and dairy cattle (32%) farmers.

Veterinarians mainly advised farmers to use antimicrobials for disease treatment (92%), but some of them also recommended AMU for preventing diseases (22%) and for growth promotion (14%). They prescribed the largest amount of antimicrobials for the treatment of ruminants. When advising AMU to farmers, only 76% of veterinarians reported always examining the animals, while 12%, 10% and 2% usually, rarely, or never did so, respectively. Most veterinarians preferred individual treatment, but some of them reported always advising the farmers to treat all animals in the pen (20%) or on the farm (14%) in the event of a disease. The decision between individual and group treatment depended on both the animals’ species (reported by 82% of veterinarians) and age (reported by 78%).

Among veterinarians, 16% believed that antimicrobials were commonly used to improve animal growth or production, and 12% believed that they were important for this purpose. These proportions were higher among veterinary pharmacy personnel, where 20% and 28% believed that antimicrobials are commonly used and important for this purpose, respectively.

Only a few feed mill personnel provided information about the antimicrobials most commonly mixed in feed by them. These included florfenicol for pigs (one feed mill), doxycycline for layer chickens (one feed mill), and the combination of streptomycin, erythromycin, oxytetracycline and colistin for layer chickens (five feed mills), with the aim of preventing gastrointestinal or respiratory infections in the animals. Other participants refused to provide details about the drugs used due to concerns about other feed mills learning this information.

When participants were asked about their experience with the efficacy of antimicrobials, 36% of farmers and 39% of veterinarians thought that antimicrobials are slightly or much less effective than previously, or that they are only effective at higher doses. Similarly, around one-third (35%) of feed mill personnel believed that the efficacy of antimicrobials has decreased based on their experience. The proportion of respondents experiencing decreased efficacy was highest among veterinary pharmacy personnel (44%).

### 2.7. Impact of the COVID-19 Pandemic

The COVID-19 pandemic tested healthcare systems, in both human and veterinary medicine, exposing vulnerabilities in access to veterinary services, diagnostics and medicine supply chains. The most commonly reported problem that farmers encountered during or after the lockdown due to the COVID-19 pandemic was related to accessing veterinary support (reported by 11%). A few farmers also had problems accessing antimicrobials (4%), disinfectants (4%), and vaccines (4%), or had to use expired antimicrobials (1%). Increased mortality was observed by 2%, and 5% had to use antimicrobials either in increased doses or more frequently than usual.

Among veterinarians and veterinary pharmacy personnel, a higher proportion of respondents experienced difficulties during the pandemic. Around one quarter (23%) of veterinarians had problems accessing farms, and one-fifth had problems accessing antimicrobials (19%), disinfectants (20%), or vaccines (20%). Increased mortality was reported by 12%, and 21% had to increase either the frequency or dose of antimicrobial treatments. Veterinary pharmacy personnel also reported difficulties with accessing antimicrobials (26%), disinfectants (24%) and vaccines (24%). A few of them (2%) were also forced to sell expired antimicrobials. Among all surveyed feed mill personnel, 16% had difficulties accessing farms (12 of 75 participants), while among those who were preparing feed containing antimicrobials, 10% had problems accessing antimicrobials and were forced to sell expired drugs (3 of 31 participants).

## 3. Discussion

To the best of our knowledge, this is the first large-scale study to provide insights into AMU practices and awareness of AMR among livestock sector stakeholders in seven former Soviet countries: Armenia, Azerbaijan, Georgia, Kazakhstan, Kyrgyzstan, Tajikistan, and Ukraine. As a KAP study, the findings primarily reflect self-reported perceptions and behaviours and do not directly measure actual AMU. Indeed, increased knowledge does not automatically translate into behaviour change, and AMU practices are influenced by broader structural, economic and regulatory factors. Nevertheless, KAP assessments remain a pragmatic and widely used approach in settings where routine AMU surveillance data are absent or limited, particularly for informing the design of targeted training, awareness-raising and stewardship interventions. In this context, the present study provides an essential baseline for identifying misconceptions, behavioural drivers and system-level constraints relevant to antimicrobial stewardship in the livestock sector of the target countries.

Through face-to-face interviews with more than 3000 participants, including farmers of priority livestock production systems, veterinarians, veterinary pharmacy personnel, and feed mill personnel, common strengths and gaps in their KAP related to AMU and AMR were revealed. Although the number of feed mills producing feed with antimicrobials was limited, their inclusion provides valuable insight into an often under-documented segment of the veterinary antimicrobial supply chain. The results of this study complement previous research conducted on AMU practices in the human health sector and among the general public, and studies assessing the prevalence of AMR in both humans and animals in the region. Table 5 summarizes the key findings of the study that are discussed in the following paragraphs in more detail. The table also includes possible system-level explanations of the findings, as well as similarities with the human health sector in the target countries.

One notable observation of this study regarding AMU practices is the fact that farm owners (and not veterinarians) were most likely to decide on AMU on their farms, and that they commonly sought advice from veterinary pharmacists (instead of field veterinarians), or relied on product labels and their own experience. One of the explanations for this finding is the difficulty in accessing veterinary services, which has been previously reported in LMICs [37], and which is perceived as an important barrier for prudent AMU in animal healthcare [3]. Obstacles to accessing veterinary care in LMICs may include long travel time, economic challenges, and regulatory hurdles. In terms of average travel time, it is more common in LMICs than in HICs that food-producing animals are located over an hour by motorized transport from veterinary practices. There is a common lack of veterinary practices in rural areas, which can be explained by veterinarians’ preference for practising in urban areas due to better work–life balance, a higher number of customers who can afford their services, better infrastructure and amenities, and more opportunities for collaboration and professional growth. Besides travel time, economic challenges can also affect farmers, especially smallholders, who may have difficulties in affording veterinary services [37]. It was also reported in our study by both veterinarians and farmers that the lockdown during the COVID-19 pandemic was an additional obstacle to the provision of on-farm veterinary services. It should also be noted that the advice on AMU from veterinary pharmacists may come with a potential conflict of interest, as it has been reported previously that pharmacists can feel pressure from pharmacy owners and the pharmaceutical industry to sell antimicrobials, and they might also have financial incentives to sell certain types of drugs [38].

Besides cost and accessibility, additional barriers to accessing veterinary care may include the lack of or insufficient veterinary–client communication, lack of trust, lack of client education, cultural beliefs, and language barriers. Among these, effective communication is recognized as an important skill for veterinarians to have, which can impact the willingness of farmers to seek veterinary advice and to uptake their recommendations [39]. Lack of trust towards doctors has also been described in studies from the region focusing on human health care. Around one-fifth of participants in a Georgian study reported not trusting their doctor when they did (24%) or did not (20%) prescribe antimicrobials for them [40]. In Armenia, lack of trust was reported by 34% of participants when doctors prescribed antimicrobials for them, and by 15% when the doctors did not prescribe these drugs. It was also mentioned by 36% of participants that doctors often prescribed antimicrobials to fulfil patient expectations, and 28% reported that they would indeed go to another doctor when disagreeing with the doctor’s decision not to prescribe antimicrobials [41]. These factors may also affect the farmer–veterinarian relationship, and thus the willingness of farmers to seek and follow the advice of veterinarians regarding AMU. It has indeed been suggested that the nature of the farmer–veterinarian relationship is an important factor for antimicrobial stewardship, and that if veterinarians are less confident in this situation, they may prescribe more antimicrobials to avoid conflicts and displeasing farmers [42].

One of the most critical findings of this study is the discrepancy between self-reported prescription use by farmers and the low frequency with which veterinary pharmacy personnel and feed mill personnel reported requiring prescriptions when dispensing antimicrobials (or feed containing antimicrobials). While 60% of farmers reported always and 19% often having a prescription when buying such drugs, 64% of veterinary pharmacy personnel never or rarely required a prescription, and 35% of feed mill personnel never required one either. At the time of the interviews, many of the target countries did not have regulations on prescription use for veterinary antimicrobials; however, these have now been widely introduced [30]. It is important to note, though, that over-the-counter (OTC) sales of antimicrobials may also happen when such regulations are in place, but there is no proper enforcement, as is reported from the human health sector. Selling antimicrobials OTC, upon a patient’s request and without a prescription from a doctor, has been reported as a common practice in many developing countries [43], including the countries in this study [20,38,40,41,43,44,45,46]. The use of OTC antimicrobials may be driven by a lack of access to qualified healthcare professionals and adequate diagnostic tools, the latter of which also favours the large-scale empirical use of antimicrobials [3] and the use of broad-spectrum antimicrobials [38]. Self-medication with antimicrobials—such as the use of leftovers, sharing drugs with family members, friends or neighbours, or purchasing drugs OTC or from abroad—may be more common in developing countries due to limited access to healthcare, the increased availability of drugs without prescription, the lack of regulations or enforcement, and the higher prevalence of communicable diseases. Other factors that may promote self-medication include those that discourage patients from visiting medical practitioners, such as the lack of time, high costs, or the lack of trust, and the convenience of obtaining antimicrobials OTC [44,46]. In this study, with regard to the livestock sector, the difficulty in accessing veterinary services described above is a factor that can affect prescription use practices. Other similarities between OTC use in the human and animal health sectors can also be seen, such as the reports of farmers using leftovers and antimicrobials received from other farmers [47].

The non-therapeutic use of antimicrobials in food-producing animals for growth promotion and disease prevention is a serious concern for public health due to its impact on AMR development [1]. Both practices have been reported by various stakeholders in this study, and thus identify another area for improvement. Among farmers, prophylactic AMU was most common in poultry production, while growth-promoting use was mainly reported by backyard and dairy cattle farmers, with a total of 75 participants (4% of all farmers) revealing such use. Among veterinarians, 22% recommended AMU for disease prevention and 14% for growth promotion. Growth promotion was perceived as an important purpose of AMU by 12% of veterinarians and 28% of veterinary pharmacy personnel. Although the proportion of farmers reporting growth-promoting use was relatively low, this finding should be interpreted with caution, as social desirability bias and under-reporting cannot be excluded, particularly in contexts where such practices are increasingly discouraged or regulated.

Importantly, the reported use of HPCIAs, particularly on chicken farms, suggests that these drugs are often used as routine first-line options rather than being reserved for exceptional cases. Fluoroquinolones, third- and fourth-generation cephalosporins, and polymyxins are critically important for human medicine, and thus their veterinary use should be restricted to those cases when less valuable antimicrobials could not be clinically effective, and their use should be based on AST whenever possible [48]. The use of broad-spectrum antimicrobials may be favoured due to factors such as diagnostic uncertainty, these drugs being perceived as highly effective, and the prioritization of individual patient needs [49]. Among these factors, the availability of laboratory diagnostic services can strongly affect the feasibility of targeted antimicrobial treatment. In our study, although many veterinarians considered laboratory testing important in the case of both infectious disease outbreaks and single infections, only 62% of all veterinarians reported having good access to a veterinary diagnostic laboratory. The lack of laboratory diagnostic capacity has been previously reported as a challenge for antimicrobial stewardship in LMICs [4]. In addition, the equipment available in veterinary laboratories in LMICs is generally limited, and improper maintenance and calibration are more common than in HICs. The barriers to proper maintenance include financial limitations and the lack of services available at the local level [50]. High costs of laboratory consumables, long procurement lead times, and the expiry of reagents upon arrival have also been reported as barriers to laboratory diagnostics in LMICs [51]. Another factor that can affect drug choices is the availability of antimicrobials on the market. In this study, all stakeholders (farmers, veterinarians, veterinary pharmacy personnel, and feed mill personnel) reported having problems with accessing antimicrobials during the COVID-19 pandemic.

An important aspect of prudent AMU is the use of antimicrobials according to the prescribed dose, administration route, and treatment duration. Almost half (47%) of the farmers interviewed in this study had a misbelief that antimicrobial treatment can be stopped when the animals’ symptoms are improving (although 91% of farmers reported following the veterinarian’s advice about the applied dose and treatment duration). While there is an increasing attention to the effectiveness of shortened antimicrobial treatment courses, such decisions need to be evidence-based and made by healthcare professionals. Non-adherence to the prescribed therapy, on the other hand, can reduce treatment effectiveness and increase the risk of relapse [52]. The findings of our study about farmers’ misbeliefs are in accordance with studies among the general population from the same region. Around one-fifth (21%) of respondents from Georgia [40], and 33% of participants from Kazakhstan [43] believed that antimicrobial treatment could be discontinued when the symptoms improved. Surprisingly, public health and medical students in Georgia also reported that they stop taking antimicrobials when their symptoms improve (32% of respondents) [53]. Similarly, 41% of adults from Yerevan, Armenia, reported discontinuing antimicrobial therapy when starting to feel better. A higher education level of participants was associated with lower odds of misusing antimicrobials in this study [41]. Emphasizing the importance of proper treatment duration in reducing the risk of AMR development could be an important component in future training initiatives for farmers and the general public in the region. As an initial step towards this direction, misconceptions revealed during our study were clarified by trained interviewers, and awareness-raising leaflets on AMR were distributed to study participants.

Veterinarians have a key role as advisers to farmers, and they need to balance animal welfare, agricultural economics and public health when it comes to the use of antimicrobials in animals [1]. Veterinarians should advocate for and educate farmers on antimicrobial stewardship, and thus they should have proper knowledge on AMR, prudent AMU, and antimicrobial stewardship programmes [54,55]. However, studies conducted among veterinary students around the globe have revealed that they commonly have knowledge gaps and express the need for more education on these topics [56,57,58,59,60,61]. FAO has also conducted university curriculum assessments on learning outcomes related to AMR and AMU in veterinary institutions of former Soviet countries, finding gaps in the education of veterinary students [62,63,64,65,66,67,68,69,70]. Similarly, in this study, most, but not all veterinarians (78%) and veterinary pharmacists (75%) reported having learned about AMR during their studies, but misconceptions and gaps in the KAP of veterinarians were also found. This included the lack of record-keeping on antimicrobial sales and prescriptions, believing that laboratory diagnostics were not necessary, and not examining the animals before advising AMU to farmers. In a study from Georgian hospitals that evaluated the uptake of an antimicrobial stewardship program by healthcare workers, the main barriers identified included knowledge gaps related to AMR and antimicrobial stewardship, skill gaps related to communication on AMU, and the underestimation of local infection rates. Initial doubts about the program’s effectiveness and patient acceptance were also raised [71]. These factors could also occur among veterinary specialists. It should also be noted that other factors, besides knowledge gaps, may affect the willingness of veterinarians to advocate for antimicrobial stewardship. Indeed, these findings should be interpreted in the context of structural constraints commonly faced by veterinarians in the region, including limited access to diagnostic services, economic pressures, dual roles in treatment and medicine supply, and client expectations, which may complicate the practical application of antimicrobial stewardship principles.

In terms of common misbeliefs of farmers about antibiotics, misidentifying their spectrum of activity (as part of how farmers define these drugs) is another important observation of this study. This misconception is not unique to farmers. Many individuals from Georgia believed that antibiotics were effective against viruses (55%) and that they could speed up recovery from common colds (55%) [40]. A similar observation was made among the adult population of Yerevan, Armenia, where 46% agreed about the advantage of taking antibiotics for a cold, and 43% believed that antibiotics were effective against viruses [41]. Almost all (92%) adults surveyed in Kazakhstan believed that common colds need to be treated with antibiotics, and many of them also thought that antibiotics can treat headaches (57%) and body aches (48%) [43]. The most typical reasons for taking antibiotics by adults from Almaty, Kazakhstan included the common cold (35%), cough (29%), sore throat (26%) and headache (23%) [72]. It should be noted that these misconceptions are not unique to former Soviet countries or to LMICs. Similarly to our findings, many Scottish dairy farmers believed that antimicrobials are effective against parasites and viruses, and that they had an anti-inflammatory and/or analgesic effect [73].

Another knowledge gap identified in this study was the differentiation between AMR and antimicrobial residues. Unfortunately, this may lead to the perception among farmers that antimicrobial residue avoidance measures, such as properly observing withdrawal periods, will also remove the threat of AMR. This is particularly important since the majority of farmers (85%) were aware of the concept of withdrawal times (Table 2), and most veterinarians (82%) always informed farmers about the withdrawal period when advising AMU. While the proper observance of the withdrawal period is an important component of prudent use, the purchase of antimicrobials without a prescription, their use as growth promoters, and the use of HPCIA and CIA are more relevant non-prudent antimicrobial practices with respect to combating AMR.

Other inappropriate practices of farmers identified in this study include the improper disposal of expired antimicrobials and the lack of some biosecurity measures. These gaps may be linked to the fact that more than half (56%) of the surveyed farmers did not have any educational background in animal health, animal husbandry, or other animal-related areas.

The above gaps could be targeted in the future with awareness-raising and training activities; however, it should be noted that one quarter of farmers interviewed in this study were not interested in learning more about antimicrobials. Any training and awareness-raising activity organized for them should, therefore, emphasize the health and economic benefits of prudent AMU and biosecurity practices. Applying the principles of behavioural science may also be beneficial to achieve long-term impact on farmers’ attitudes and behaviour. As noted previously, the findings of this study are not unique to former Soviet countries. Similar knowledge gaps and imprudent practices were reported in other countries, such as our study in the Western Balkans [74], and studies from other parts of Europe [73]. These underline the need for a global effort and collaboration in addressing AMR.

As discussed above, the knowledge gaps and imprudent practices presented in this study may result from various behavioural, social, economic, organizational, structural and regulatory constraints, and thus should be addressed with system-level antimicrobial stewardship interventions that take into consideration the specifics of LMICs. The challenges of implementing antimicrobial stewardship in low-resource settings, as well as best-practice examples, have been described elsewhere [75,76,77]. Findings of these studies could be used when tailoring interventions for the former Soviet countries covered in our manuscript.

The strengths of our study include the large sample size with over 3000 respondents across seven countries, the inclusion of multiple stakeholder groups for their diverse perspectives, and the wide geographic coverage of the countries involved. However, as the study is based on self-reported information, social desirability bias, recall difficulty, and the misunderstanding of questions cannot be excluded. Nevertheless, the voluntary nature of the study, the confirmed anonymity of respondents, and the explanations provided by trained interviewers aimed to minimize the risk of these biases. Differences in sampling intensity may also limit direct comparability across countries. In addition, as a limitation of the manuscript, it should be noted that more information on AMU and AMR is published from the human health sector than from livestock in the target countries, and some of the references are older than 5 years, which poses a limitation to the interpretation and comparability when citing these studies.

## 4. Materials and Methods

The study received ethics approval from the Research Ethics Boards (REB) of the University of Guelph, Canada (approval number: 20-05-010). The surveys and related materials (such as field instructions and informed consent forms) were developed by FAO as described by Kovacs et al. [74]. The study was performed with the support of national authorities, non-governmental organizations, and national AMR/AMU experts. The interviews were conducted by the Strategic Development Agency (SDA) in Armenia; the Consulting Group LLC, in collaboration with the Food Safety Agency (AFSA) in Azerbaijan; the National Animal Identification and Traceability System (NAITS) project team of FAO in Georgia; the Republican Public Association “Kazakhstan Congress of Veterinary” in Kazakhstan; the Rural Areas Development Initiative (RADI) and the National Pasture Users’ Association of Kyrgyzstan “Kyrgyz Jayity” in Kyrgyzstan; the Association of Veterinarians of Tajikistan (TVA) in Tajikistan; and the Association of Ukrainian Pig Breeders in Ukraine. The online data collection platform (KoboCollect) was managed by SDA for each country.

### 4.1. Study Design and Population

Survey participants included farmers of priority livestock species, field veterinarians (providing services to farmers), veterinary pharmacy personnel, and feed mill personnel. The list and number of participants to be interviewed per stakeholder group and per region were determined with an emphasis on the main production systems in each country based on animal inventory data and information from national authorities and local experts. The regions covered in the study for each country are shown in Table 6, and in Supplementary Materials Document S2 (on maps). If the pre-defined interview numbers could not be reached for one group or region, additional interviews were conducted with other stakeholders to achieve the expected national total. This is particularly applicable to commercial feed mills, since they were present in small numbers in some of the participating countries. A prerequisite for respondents was to have relevant knowledge about AMU policies and practices at the farm, veterinary practice, pharmacy, or feed mill. All respondents signed a written informed consent form before participating in the study.

The questionnaires used in this study were developed by FAO and recently described in detail by Kovacs et al. [74]. In brief, a series of tailored questionnaires was used for different stakeholder groups to assess their KAP related to AMU and AMR. Topics included the sources of antimicrobials, prescription use, growth promotion with antimicrobials, withdrawal periods, handling of expired drugs, and treatment practices. The antimicrobials most used (by farmers), prescribed (by veterinarians), or sold (by pharmacies and feed mills) were identified using country-specific coded lists with product images and names. The surveys also gathered information about on-farm practices related to record-keeping, disease management, diagnostics, hygiene, and biosecurity. The impact of the COVID-19 pandemic on animal health, veterinary service access, and medicine availability was also captured. Question formats included multiple-choice, ranking, matrix, and free-text options, with conditional follow-up questions in some cases. Quantitative AMU data were not collected. The questionnaires and survey instructions are available in Supplementary Materials Document S1. The survey process, which included the translation of questionnaires to the local languages, the training of enumerators, and the pre-testing of questionnaires, has also been described recently by Kovacs et al. [74]. Briefly, enumerators were trained on survey implementation in each country based on the survey instructions developed by FAO (Supplementary Materials Document S1). In each country, before implementation, the questionnaires were reviewed by national experts familiar with the livestock sector to ensure relevance and proper phrasing. Pre-implementation testing of the questionnaires was also performed in each country with a small number of participants from each stakeholder group.

All surveys were conducted face-to-face, and most of them took place between December 2020 and July 2022, except for Kazakhstan, where the interviews took place between April and May 2025 (Table 6). In addition, there were two rounds of surveys in Kyrgyzstan, with the first round being implemented between September 2020 and April 2021 (covering farmers, veterinarians and veterinary pharmacies), and the second round being conducted between September and October 2023 (covering only farmers, except beekeepers). For Kyrgyzstan, the manuscript includes the results of the 2020–2021 surveys for beekeepers, veterinarians and veterinary pharmacies, and the results of the 2023 surveys for all other farmer groups. Participants were informed that their data and responses would be treated anonymously and confidentially, and that they could skip any questions, but they were encouraged not to do so. After the completion of surveys, enumerators discussed and clarified misconceptions that were revealed during the interviews, and provided an information leaflet to participants about AMU and AMR (https://openknowledge.fao.org/handle/20.500.14283/cb1585en (accessed on 22 August 2025)).

### 4.2. Data Analysis

Data were extracted from KoboCollect in Microsoft Excel format, which was then used for data cleaning and analysis. Duplications and errors were removed during data cleaning, while incomplete surveys were kept in the datasets. Data analysis was performed in Microsoft Excel Version 2506 and R Core Team 4.2.1. (2022).

## 5. Conclusions

Antimicrobial resistance is a global threat to human, animal and environmental health, which may disproportionately affect LMICs. To the best of our knowledge, this was the first large-scale study that aimed to assess the knowledge, attitudes, and practices of livestock sector stakeholders in former Soviet countries to identify misconceptions and inappropriate practices related to antimicrobial use and resistance. Detailed country reports with the findings and recommendations were shared with participating countries, and are available on FAO’s website (https://www.fao.org/antimicrobial-resistance/resources/publications-archive/en/ (accessed on 3 February 2026)) [32,33,34,35,36], supporting the translation of the study results into national antimicrobial stewardship actions. The areas for improvement identified through this regional synthesis highlight the need for strategies that extend beyond farmer trainings and systematically address veterinary service delivery, access to diagnostics, regulation and their enforcement related to antimicrobial drug distribution, and the roles of veterinary pharmacies and feed mills in the antimicrobial supply chain. While significant progress has already been made in the target countries, such as the introduction of regulations on prescription use for veterinary antimicrobials, further interventions with systems-level approaches are essential for achieving sustainable reductions in imprudent antimicrobial use and limiting the development and spread of antimicrobial resistance in the region. Such antimicrobial stewardship interventions should be developed taking into consideration the specific context of LMICs.

## Figures and Tables

**Table 1:** Number of interviews performed.

Country	Total # Participants	Interviews Performed										
		F	*DC*	*BC*	*SR*	*C*	*P*	*B*	*BY*	V	VP	FM
Armenia	613	452	- *	- *	- *	*100*	*100*	*100*	*200* *	100	51	10
Azerbaijan	302	192	*105*	-	*4*	*10*	-	-	*71*	-	110	-
Georgia	456	423	*4*	-	*21*	*3*	*2*	*26*	*394*	21	10	2
Kazakhstan	427	261	*34*	*39*	*31*	*25*	-	-	*178*	107	36	23
Kyrgyzstan	422	221	*100*	*100*	*100*	*203*	-	*12*	*100*	101	100	-
Tajikistan	288	61	*111*	*107*	*111*	*82*	-	*100*	*92*	103	112	12
Ukraine	504	335	*46*	*18*	*30*	*82*	*51*	*64*	*44*	74	67	28
Total	3012	1945	*400*	*264*	*297*	*505*	*153*	*302*	*879*	506	486	75

F: farmers, *DC*: dairy cattle section, *BC*: beef cattle section, *SR*: small ruminant section, *C*: chicken section, *P*: pig section, *B*: bee section, *BY*: backyard section, V: veterinarians, VP: veterinary pharmacy personnel, and FM: feed mill personnel. Numbers in italics indicate the species-specific surveys completed by farmers. The total number of species-specific surveys completed is greater than the number of farmers who participated (column F), as they were requested to complete a separate survey for each species raised for income. * Since there were no questionnaires specific to cattle and small ruminant farms available at the time of the surveys in Armenia, these farms were interviewed according to the backyard survey template.

**Table 3:** The 15 most used antimicrobials reported by farmers of different production systems.

Antimicrobial	WHO Category	Dairy Cattle Farmers (n = 287)	Beef Cattle Farmers (n = 169)	Small Ruminant Farmers (n = 124)	Chicken Farmers (n = 276)	Pig Farmers (n = 72)	Bee-keepers (n = 106)	Backyard farmers (n = 301)
Ceftiofur	HPCIA	5.6%	1.2%	0.8%	-	-	-	0.7%
Colistin	HPCIA	-	-	-	2.5%	8.3%	-	1.0%
Enrofloxacin	HPCIA	2.4%	5.3%	1.6%	**17.8%**	2.8%	0.9%	4.0%
Enrofloxacin + colistin	HPCIA	0.7%	1.2%	1.6%	4.7%	-	-	1.7%
Azithromycin	CIA	3.8%	4.7%	6.5%	6.5%	-	-	2.3%
Gentamicin	CIA	5.6%	3.0%	4.0%	7.6%	-	-	3.0%
Streptomycin	CIA	5.9%	1.8%	1.6%	1.8%	1.4%	3.8%	2.7%
Tylosin	CIA	5.2%	3.6%	4.0%	2.2%	19.4%	0.9%	9.3%
Benzylpenicillin + (dihydro)-streptomycin	HIA + CIA	6.3%	15.4%	6.5%	0.4%	18.1%	-	9.0%
Amoxicillin	HIA	11.1%	3.0%	12.1%	4.0%	**27.8%**	1.9%	9.3%
Ampicillin	HIA	0.7%	3.6%	6.5%	4.0%	-	0.9%	2.0%
Benzylpenicillin	HIA	9.1%	9.5%	12.1%	6.9%	-	0.9%	14.3%
Doxycycline	HIA	2.4%	2.4%	4.0%	10.1%	4.2%	5.7%	1.0%
Oxytetracycline	HIA	**20.6%**	**34.9%**	**25.8%**	6.5%	11.1%	**57.5%**	**20.3%**
Sulfadiazine + trimethoprim	HIA	1.0%	1.8%	2.4%	1.4%	-	0.9%	1.0%

WHO Category: categorization of antimicrobials based on the World Health Organization (WHO) Medically Important Antimicrobials List for Human Medicine. HPCIA: highest priority critically important antimicrobials, CIA: critically important antimicrobials, HIA: highly important antimicrobials [15]. The most used antimicrobial by each farmer group is shown in bold.

**Table 4:** The 15 most commonly prescribed or sold antimicrobials reported by veterinarians and veterinary pharmacy personnel, respectively.

Antimicrobial	WHO Category	Veterinarians (n = 389)	Veterinary Pharmacy Personnel (n = 359)
Ceftiofur	HPCIA	2.8%	1.1%
Ciprofloxacin	HPCIA	3.6%	1.4%
Enrofloxacin	HPCIA	3.9%	5.3%
Azithromycin	CIA	2.6%	0.3%
Gentamicin	CIA	1.0%	1.9%
Streptomycin	CIA	0.0%	9.2%
Tylosin	CIA	7.5%	7.5%
Benzylpenicillin + (dihydro)streptomycin	HIA + CIA	19.3%	6.7%
Benzylpenicillin + neomycin	HIA + CIA	2.6%	5.3%
Amoxicillin	HIA	14.7%	10.6%
Benzylpenicillin	HIA	5.4%	6.7%
Chlortetracycline	HIA	1.8%	2.8%
Florfenicol	HIA	2.8%	0.6%
Oxytetracycline	HIA	**20.8%**	**30.9%**
Sulfadiazine + trimethoprim	HIA	2.6%	1.9%

WHO Category: categorization of antimicrobials based on the World Health Organization (WHO) Medically Important Antimicrobials List for Human Medicine. HPCIA: highest priority critically important antimicrobials, CIA: critically important antimicrobials, HIA: highly important antimicrobials [15]. The most commonly prescribed or sold antimicrobial by each stakeholder group is shown in bold.

**Table 5:** Key findings of the study covered in the discussion.

Stakeholder Group	Key Knowledge Gaps/ Imprudent Practices	Possible System-Level Explanations	Similarities with the Human Health Sector
Farmers	Farm owners deciding on antimicrobial use on their farms, based on product labels, their own experience, or advice from veterinary pharmacists (without consulting veterinarians for advice)	- Difficulty in accessing veterinary services - Poor farmer-veterinary relationships	- Lack of trust towards doctors, disagreement between patients and doctors
Veterinary pharmacy and feed mill personnel	Veterinary pharmacy and feed mill personnel selling antimicrobials or feed containing antimicrobials without requiring a prescription	- Lack of regulation or enforcement on prescription requirements	- Selling antimicrobials over-the-counter in human pharmacies
Farmers and veterinarians	Non-therapeutic use of antimicrobials for growth promotion	- Lack of regulation or enforcement	-
Chicken farmers	Common use of highest priority critically important antimicrobials	- Diagnostic uncertainty, poor access to diagnostic laboratories - Limited market availability of antimicrobials	- Barriers to time- and cost-effective laboratory diagnosis
Farmers	Misbelief about proper antimicrobial treatment duration (believing that antimicrobial treatment can be stopped when the symptoms are improving)	-	- Similar misbelief among patients
Farmers	Misbelief about the spectrum of activity of antibiotics	-	- Similar misbelief among patients
Veterinarians	Lack of education on antimicrobial resistance, misbeliefs about laboratory diagnostics not being necessary, and not examining the animals before advising antimicrobial use to farmers	- Gaps in the undergraduate education of veterinarians	-

**Table 6:** Regions covered in the study per country.

Country	Regions Covered in the Study	Study Timeframe
Armenia	Aragatsotn, Armavir, Kotayk, Syunik and Vayots Dzor marzes	March–October 2021
Azerbaijan	Agstafa, Absheron, Agdash, Aghjabadi, Agsu, Astara, Balakan, Barda, Beylagan, Gadabay, Goranboy, Goygol, Hajigabul, Ismayilli, Jalilabad, Khachmaz, Kurdamir, Lankaran, Masally, Qabala, Qazakh, Quba, Qusar, Saatly, Sabirabad, Salyan, Samukh, Shabran, Shaki, Shamakhi, Shamkir, Tovuz, and Yevlakh districts	November–December 2021
Georgia	Adjara, Guria, Imereti, Kakheti, Kvemo Kartli, Mtskheta-Mtianeti, Racha-Lechkhumi and Kvemo Svaneti, Samegrelo-Zemo Svaneti, Samtskhe-Javakheti, Shida Kartli, and Tbilisi regions	October–November 2021
Kazakhstan	Akmola, Almaty, East Kazakhstan and Turkistan oblasts	April–July 2025
Kyrgyzstan	Chui, Issyk-Kul, Jalab-Abad and Osh oblasts	September 2020–April 2021 and September–October 2023
Tajikistan	Districts of Republican Subordination, Khatlon and Sughd oblasts	February–July 2022
Ukraine	Cherkasy, Chernihiv, Chernivtsi, Dnipropetrovsk, Donetsk, Ivano-Frankivsk, Kharkiv, Kherson, Khmelnytskyi, Kirovohrad, Kyiv, Luhansk, Lviv, Mykolaiv, Odesa, Poltava, Rivne, Sumy, Ternopil, Vinnytsia, Volyn, Zakarpattia, Zaporizhzhia, and Zhytomyr oblasts	December 2020–April 2021

## Data Availability

The original contributions presented in this study are included in the article/Supplementary Materials. Further inquiries can be directed to the corresponding author.

## Supplementary Materials

The following supporting information can be downloaded at https://www.mdpi.com/article/10.3390/antibiotics15040384/s1. Document S1: Survey instructions and questionnaires; Document S2: Regions covered by the study on maps.

## Abbreviations

The following abbreviations are used in this manuscript:

AMR	Antimicrobial Resistance
AMU	Antimicrobial Use
AST	Antimicrobial Susceptibility Testing
CIA	Critically Important Antimicrobials
FAO	Food and Agriculture Organization of the United Nations
FAO-PMP-AMR	FAO Progressive Management Pathway for Antimicrobial Resistance
GLASS	Global Antimicrobial Resistance and Use Surveillance System
HIA	Highly Important Antimicrobials
HICs	High-income Countries
HPCIA	Highest Priority Critically Important Antimicrobials
InFARM	International FAO Antimicrobial Resistance Monitoring System
KAP	Knowledge, Attitudes, and Practices
LMICs	Low- and Middle-Income Countries
MDR	Multidrug-resistant
NAPs	National Action Plans
OTC	Over-the-counter
UNEP	United Nations Environment Programme
WHO	World Health Organization
WOAH	World Organization for Animal Health
