# Supplementary material for: Knowledge, Attitudes and Practices Related to Antimicrobial Use and Resistance Among Livestock Sector Stakeholders in Seven Former Soviet Countries: A Multi-Country Regional Analysis
Source: Antibiotics (Basel). 2026 Apr 9;15(4):384. doi: 10.3390/antibiotics15040384 (PMC13113907; doi:10.3390/antibiotics15040384)
Supplement: Supplementary file 1 [file antibiotics-15-00384-s001.zip › Document S2. Regions covered by the study on maps_supplementary.pdf]

Regions covered by the study on maps

Armenia

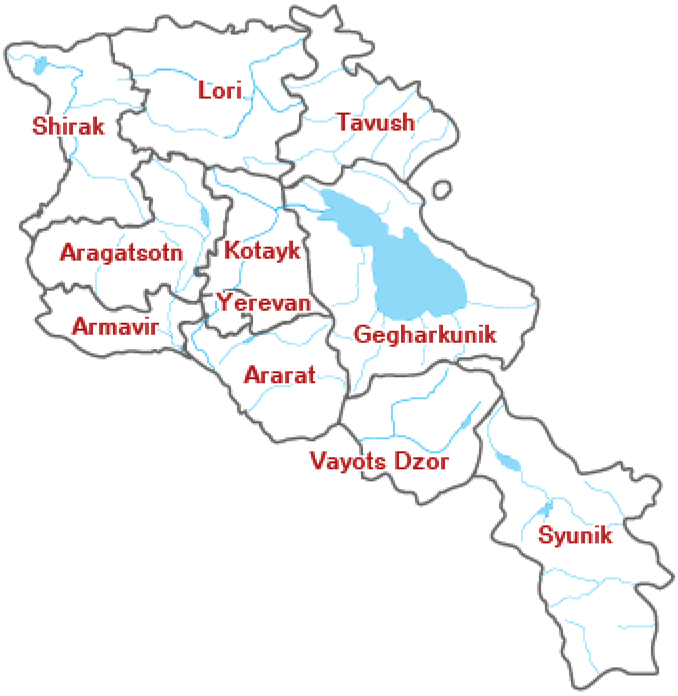

Aragatsotn, Armavir, Kotayk, Syunik and Vayots Dzor marzes

Azerbaijan

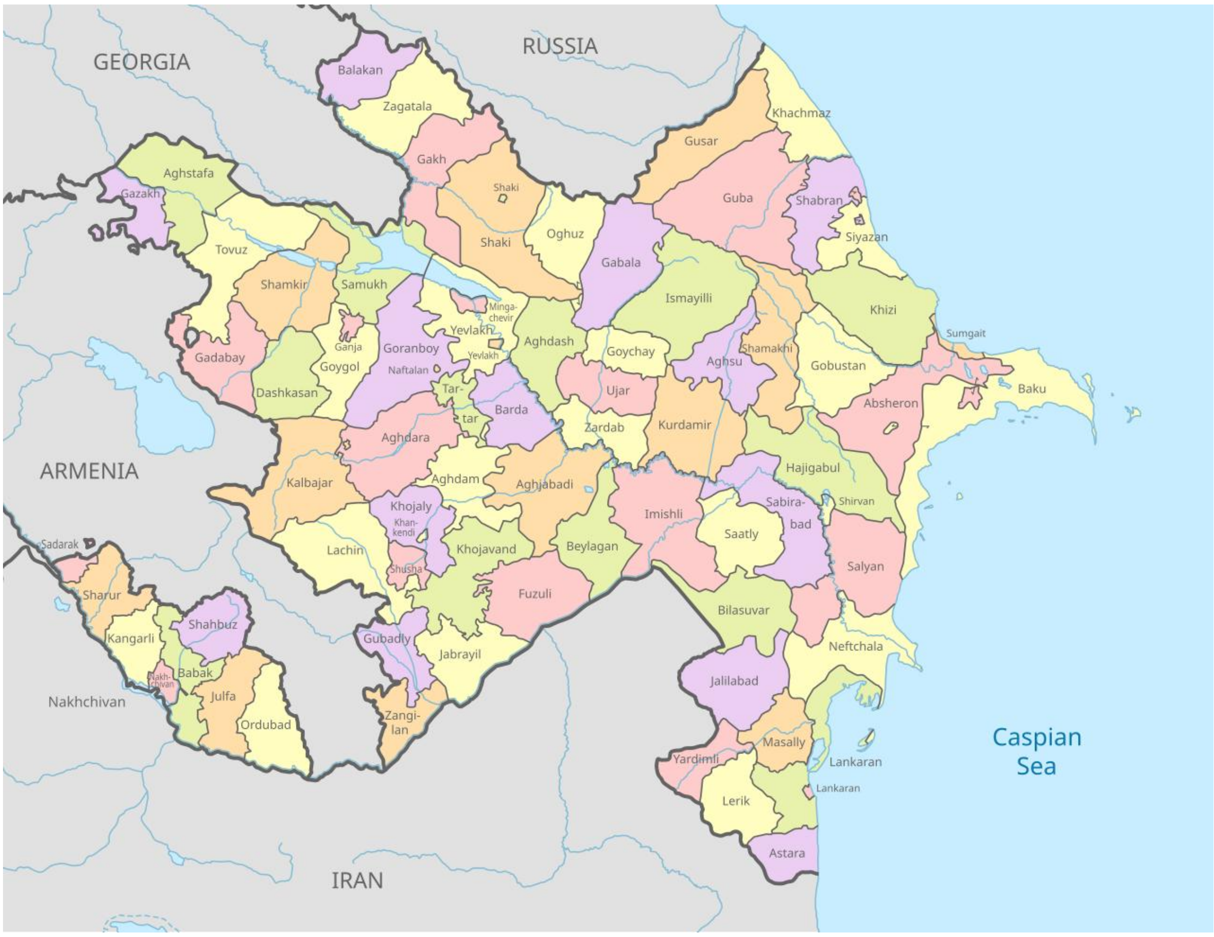

Agstafa, Absheron, Agdash, Aghjabadi, Agsu, Astara, Balakan, Barda, Bey-lagan, Gadabay, Goranboy, Goygol, Hajigabul, Ismayilli, Jalilabad, Khach-maz, Kurdamir, Lankaran, Masally, Qabala, Qazakh, Quba, Qusar, Saatly, Sabirabad, Salyan, Samukh, Shabran, Shaki, Shamakhi, Shamkir, Tovuz, and Yevlakh districts

Georgia

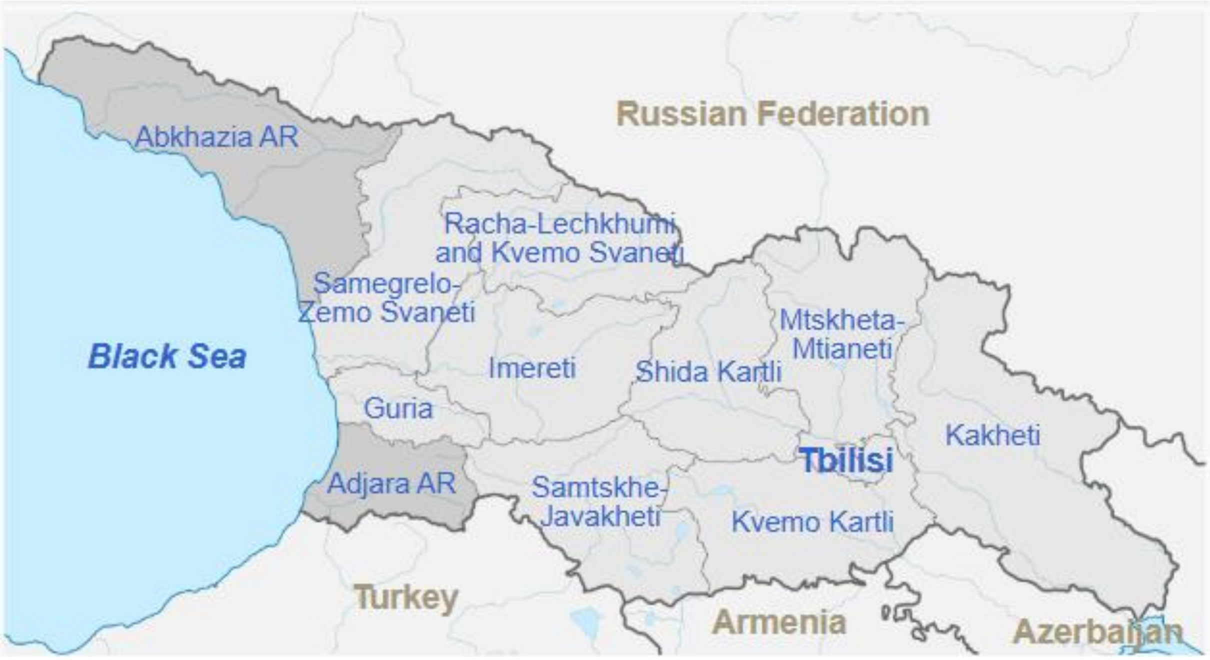

Adjara, Guria, Imereti, Kakheti, Kvemo Kartli, Mtskheta-Mtianeti, Racha-Lechkhumi and Kvemo Svaneti, Samegrelo-Zemo Svaneti, Samtskhe-Javakheti, Shida Kartli, Tbilisi regions

Kazakhstan

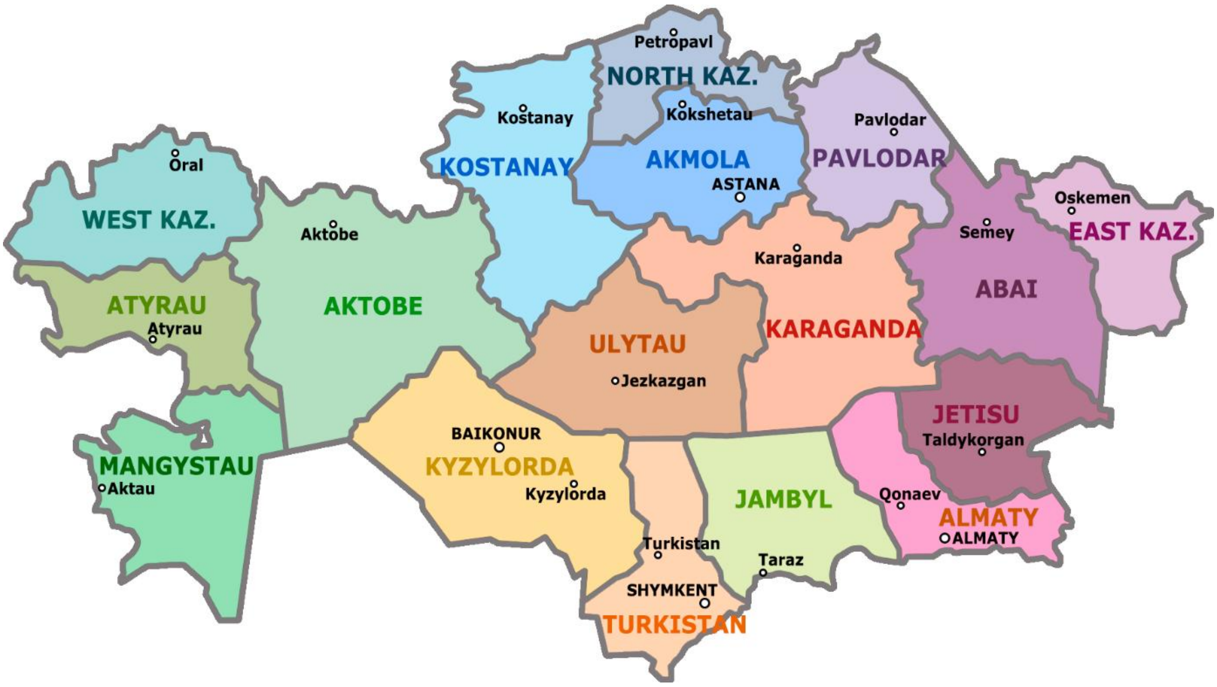

Akmola, Almaty, East-Kazakhstan and Turkistan oblasts

Kyrgyzstan

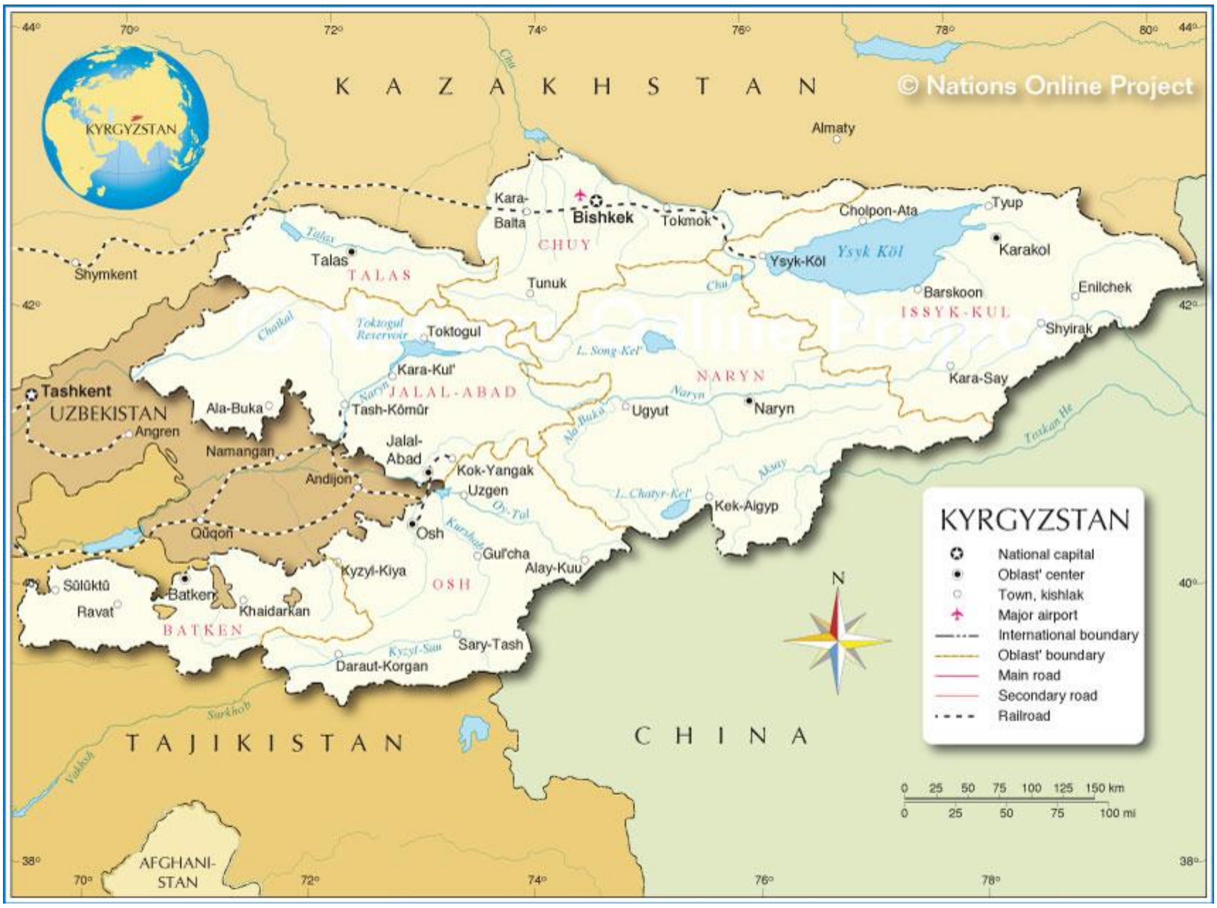

Chui, Issyk-Kul, Jalab-Abad and Osh oblasts

Tajikistan

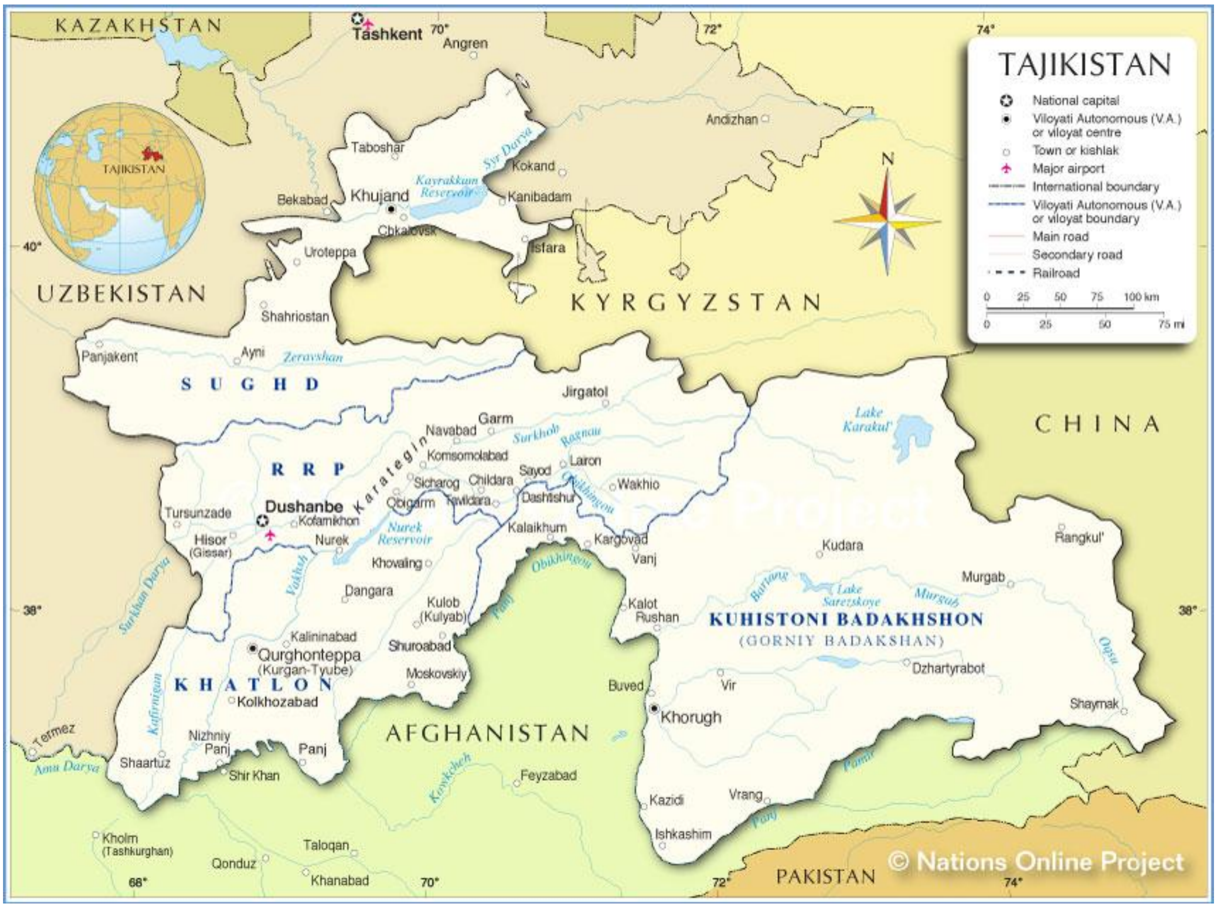

Districts of Republican Subordination, Khatlon and Sughd oblasts

Ukraine

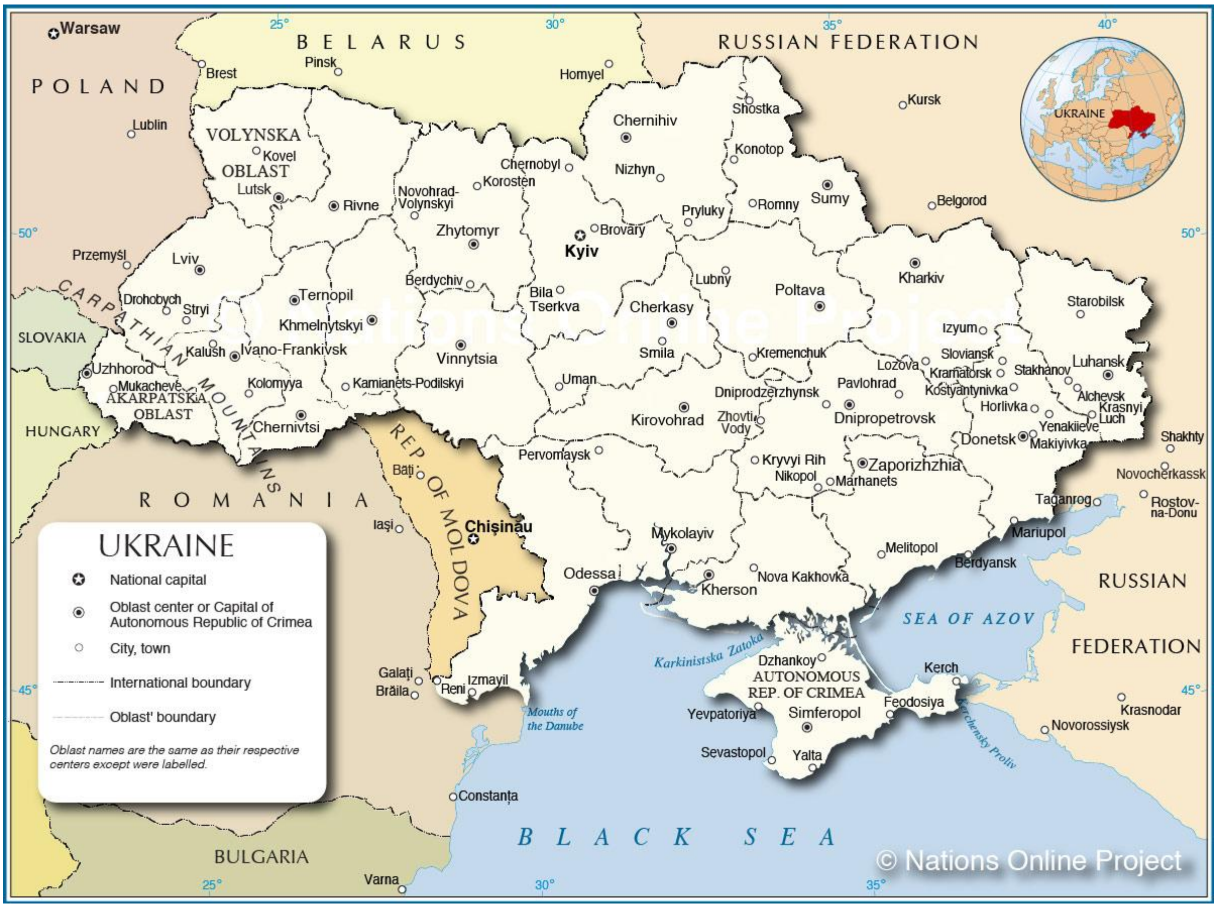

Cherkasy, Chernihiv, Chernivtsi, Dnipropetrovsk, Donetsk, Ivano-Frankivsk, Kharkiv, Kherson, Khmelnytskyi, Kirovohrad, Kyiv, Luhansk, Lviv, Mykolaiv, Odesa, Poltava, Rivne, Sumy, Ternopil, Vinnytsia, Volyn, Zakarpattia, Za-porizhzhia, and Zhytomyr oblasts
